# Immediate pressor response to oral salt and its assessment in the clinic: a time series clinical trial

**DOI:** 10.1186/s40885-022-00209-2

**Published:** 2022-09-15

**Authors:** Sepiso K. Masenga, Leta Pilic, Benson M. Hamooya, Selestine Nzala, Douglas C. Heimburger, Wilbroad Mutale, John R. Koethe, Annet Kirabo, Sody M. Munsaka, Fernando Elijovich

**Affiliations:** 1 HAND Research Group, School of Medicine and Health Sciences, Mulungushi University, Akapelwa street, LUTH Premises, Livingstone, Zambia; 2 Department of Biomedical Sciences, School of Health Sciences, University of Zambia, Lusaka, Zambia; 3 Faculty of Sport, Health and Applied Science, St. Mary’s University, Twickenham, London, UK; 4 School of Public Health and School of Medicine, University of Zambia, Lusaka, Zambia; 5 Department of Medicine, Vanderbilt Institute for Global Health and Vanderbilt University Medical Center, Nashville, TN USA

**Keywords:** Immediate pressor response, Blood pressure, Systolic pressure, Arterial pressure, Sodium chloride, Hypertension, Pulse pressure

## Abstract

**Background:**

High blood pressure (BP) is associated with high-salt consumption especially in sub-Saharan Africa. Although the pressor effect of salt is viewed as a chronic effect, some studies suggest that a salty meal may increase BP immediately in some individuals, and that this effect may cause endothelial dysfunction. Therefore, the aim of our research was to study the immediate pressor response to oral salt (IPROS) and its determinants, with the expectation that a simple methodology may be devised to diagnose it in the clinic or in low-resource environments.

**Methods:**

We conducted a time series trial at Livingstone Central Hospital. We present data in 127 normotensive participants who ingested 2 g of sodium chloride; their BP was monitored for 120 minutes in intervals of 10 minutes. Sociodemographic and clinical data were collected. Descriptive and inferential statistics were used for analyses of data.

**Results:**

Median age was 30 years (interquartile range, 22–46 years) and 52% were female patients. An increase of ≥10 mmHg in mean arterial pressure (MAP), considered a clinically significant IPROS, was present in 62% of participants. Systolic BP 30 minutes after the salt load was a significant predictor of IPROS, avoiding the need to calculate MAP in the clinic setting.

**Conclusions:**

We confirm the presence of an IPROS in a high proportion (62%) of otherwise normotensive participants. The average time course for this response was 30 minutes and its duration was sustained for the 120-minutes period of study in most of the participants. Prediction of IPROS by ∆SBP (change in systolic blood pressure) at 30 minutes allows for easy assessment of possible responder status in the clinic. Our data indicate that the IPROS to oral salt-loads in the range currently consumed by the Western world and African populations in single meals may increase the 24-hour BP load, which is a risk factor for hypertension and target organ damage. The relevance of our findings indicates the need to include dietary sodium assessment in the diagnosis, prevention, and management of high BP.

**Supplementary Information:**

The online version contains supplementary material available at 10.1186/s40885-022-00209-2.

## Background

Salt sensitivity of blood pressure (SSBP) is a response of blood pressure (BP) that parallels changes in salt intake [1]. It is a risk factor for future hypertension, end-organ damage, and cardiovascular disease (CVD) [1, 2] and is more common in blacks [1]. Changes in BP due to high-salt intake are associated with functional changes in the vasculature. This may promote hypertension in the long term [2] in normotensive participants.

Because SSBP is normally distributed in the population, its diagnosis is made by arbitrary cutoffs in the magnitude of the BP response to salt loading or salt depletion, which are achieved by days to weeks of dietary intervention [1, 3] or with a 3-day inpatient protocol developed decades ago [4, 5]. Generally accepted cutoffs have been an increase of ≥10% or of 10 mmHg in mean arterial pressure (MAP) after salt loading [1, 6–9]. Those individuals who do not sustain such magnitude of change in MAP after salt loading are classified as salt resistant. Systolic BP (SBP) and diastolic BP (DBP) components have also been used to diagnose SSBP, but the majority of studies use MAP, perhaps because the latter is a better indicator of tissue perfusion.

Few studies have reported an immediate effect of salt on BP. Data in black populations is lacking. Whether such response relates to conventionally defined SSBP is not known. However, there is evidence that consuming foods high in salt (≈1495 mg of sodium) suppresses brachial artery flow mediated dilatation within 30 minutes [10]. Hence, high-salt intake may impair vasodilation for an unknown duration during the postprandial periods of the day, increasing the 24-hour BP load in susceptible individuals.

In our study, we indirectly assessed vascular health by means of pulse pressure, ankle brachial index (ABIs) and the “salt blood test”. The latter is based on the fact that the glycocalyx of red blood cells (RBCs) and endothelial cells vary jointly in response to stimuli. Disruption of the endothelial glycocalyx diminishes its sodium buffering capacity by decreasing negatively charged proteoglycans. This allows for leakage of intravascular sodium to the interstitium, an abnormality that has been associated with essential hypertension. Changes in the RBC glycocalyx, parallel to those in the endothelium, can be assessed by the RBC sedimentation rate in iso-osmolar (dextran) solutions produced by mixing blood with a standard solution of low sodium concentration [11, 12]. Greater disruption of the RBC glycocalyx leads to higher erythrocyte sedimentation rates. The ratio between individual measurements and the sex-corrected mean in the population is called the erythrocyte sodium sensitivity (ESS) and it increases as the vascular dysfunction of a subject increases [11, 13]. It has been shown that a low erythrocyte buffering capacity for sodium correlated negatively with ESS and positively with BP [13]. In contrast, the relationship between ESS and SSBP has not been evaluated.

The aims of our study were to (1) determine the immediate pressor effect of salt on BP in blacks, (2) determine the percentage of normotensive participants that sustain a clinically significant IPROS, defined as an increase in MAP of ≥10 mmHg, in response to 2 g of oral salt, (3) study the time course of such pressor response, (4) explore its correlates, including biomarkers of vascular health, and (5) investigate the ability of pre-defined increases in SBP to predict the clinically significant IPROS with timed measurements in the clinic. The study was conducted in black individuals living in the sub-Saharan African country, Zambia.

## Materials and methods

### Study design and setting

This was a time series trial where participants served as their own controls to minimize bias. The study was conducted at Livingstone Central Hospital among normotensive health volunteers, following the recommendation by other investigators that the putative genetic susceptibility to a pressor effect of salt is likely better assessed before hypertension develops, since the latter is a confounder by increasing the prevalence of salt sensitivity, probably by the effect of environmental factors [14, 15].

### Ethical approval

This study was approved by the University of Zambia Biomedical Research Ethics committee (IRB00001131 of IORG0000774) and the National Health Research Ethics Board under the reference number 981-2020. All participants signed an Institutional Review Board-approved consent form after a thorough explanation of the protocol before being included in the study.

### Recruitment rationale

In a large sample of normotensive participants studied for traditional SSBP with the 3-day inpatient protocol [4], MAP reduction owing to salt depletion was about 4.5 ± 2.5 mmHg. Therefore, we chose 10 mmHg of MAP (2 standard deviations above the mean) to define a clinically significant IPROS to 2 g of oral salt (788 mg of sodium). The 120-minute period of follow-up was chosen based on a previous study [10]. Because traditional SSBP is present in about one quarter of the normotensive population, we hypothesized that for each IPROS responder we would find three nonresponders. We therefore planned to recruit 120 participants to have at least 30 IPROS responders and describe their characteristics. Assuming a dropout rate of 10% we ultimately set a recruitment goal of 132 participants. Recruitment was carried out by publicizing the study through health talks at the outpatient departments and the medical clinic for individuals seeking health certification for employment. All normotensive adults (≥18-year-old) were eligible and were recruited if they provided both verbal and written informed consent. Participants seeking health care for acute or chronic problems in these facilities were excluded, but human immunodeficiency virus (HIV) positivity in the absence of clinical illness was allowed. After the intended recruitment of 132 participants was completed, five participants were excluded due to errors in data collection (missing BP readings). Results for the remaining 127 participants are presented here. There was no randomization step in the protocol because each participant served as his or her own control before the intervention.

The main aim of the study was to describe the prevalence of IPROS in a normotensive population, but we also explored whether SBP responses could be used as predictors of IPROS since MAPs are not routinely calculated in the clinic. Analyses of ∆SBP (change in SBP) as a predictor of IPROS were carried out by regression techniques (see Materials and methods). Age, sex, employment status, marital status, HIV status, body mass index (BMI), ABI, fasting blood sugar, RBC count, and ESS percentage were used as independent variables in univariate analyses. Measurements of pulse pressure, ABI, and the previously described “salt blood test” [13] that determines ESS were used as surrogate markers for vascular health.

Kidney and liver function results were used to potentially exclude any potential participants with kidney or liver impairment but were not intended for statistical analyses. No potential participant screened had abnormal kidney or liver function results.

### Study procedures

All participants fasted overnight for at least 8 hours prior to the study. On arrival to the research room, patients were instructed to rest in the seated position. BPs were recorded every 10 minutes for 40 minutes and the recording at minute 40 was used as the baseline rested value. BP measurements were made with an automated validated sphygmomanometer (Omron HEM-7120; Omron Healthcare co., Limited, Kyoto, Japan) in the seated position, with the participants’ arms at heart level and their feet and back supported by the floor and chair, respectively. Immediately thereafter, participants were given two tablets of sodium chloride containing 1 g (394 mg of sodium) each and BPs were monitored in 10-minute intervals for 120 minutes. Study data were collected and managed using a secure, web-based software platform, REDCap (Research Electronic Data Capture, Vanderbilt University, Nashville, TN, USA; https://projectredcap.org/) [16, 17], which allows for data auditing and exporting into statistical packages. Anthropometric, laboratory and clinical data were collected prior to the preintervention period. ABI was performed using a Sonotrax Vascular Doppler (Ultrasonic pocket doppler; Shanghai international Holding Corp. GmbH, Hamburg, Germany) with an 8 MHz vascular probe and a sphygmomanometer with attached gauge. Patients were allowed to rest supine for 5 minutes before measurement. Pulse pressure was calculated as SBP minus DBP. Calculation of ABI was performed by dividing the highest ankle SBP (measured at both the dorsalis pedis and posterior tibial arteries) by the higher brachial SBP (measured bilaterally).

Both the principal investigator and the trained research assistants who were under the supervision of the principal investigator, administered the whole protocol and interventions. Masking of study conditions was not applicable, but the research assistants were blinded to the primary and secondary outcomes.

### Rationale for using a 2 g oral salt load with free fluid intake

Despite all guidelines and recommendations to reduce it, sodium intake in Western countries is about 140–160 mmol/day and in African adults 116–142 mmol/day [18]. Hence, each of the three meals of a day will have approximately 40–50 mmol (with the heaviest meal having a higher amount). So, we chose 2 g of salt, which amounts to 787 mg or 34.2 mmol sodium as a conservative “load”, slightly lower than that of a regular meal, purposefully avoiding a nonphysiologically, excessive sodium load.

Regarding possible effects of the putative accompanying water intake on blood volume or BP, the traditional view of Guytonian physiology was that increasing salt intake would be associated with increased thirst and water intake, to restore plasma osmolality and that the associated volume-expansion would drive compensatory natriuresis. However, decades ago, this sequence of events was disputed by investigators who found body weight and volume losses during increased salt intake [19]. More recent research has confirmed these observations and shown that salt intake is associated with a catabolic state with generation of endogenous water instead [20]. Even though the current view is that fluid intake is not a significant contributor to the pressor effect of salt, we provided participants with 250 mL of water to take with the salt for the sake of consistency and uniformity, but we made no restrictions to further fluid intake or measured it.

### Statistical analysis

Data were analyzed using IBM SPSS ver. 22.0 (IBM Corp., Armonk, NY, USA). Medians and frequency distributions were used to represent descriptive data. Comparison of BPs among multiple time intervals was carried out with the Friedman test with the Dunn method for post-hoc contrasting of means between each time interval and baseline.

Sample proportion summary hypothesis test (StatCrunch, Pearson Education, London, UK) was used with the proportion of IPROS responders observed at each 10-minute intervals to test at which time point half of the responders would exhibit the defining increase in MAP of ≥10 mmHg with alpha < 0.05. The Standard-Wald method was used to compute 95% confidence limits of proportions.

Chi-square testing was employed to analyze the distribution of ESS groups in IPROS responders versus nonresponders. Univariable and multivariable logistic regression models were used to investigate the best timing for the best ∆SBP prediction of IPROS. Multivariable logistic regression was also used to assess the relationship between ESS and IPROS, controlling for possible confounders (age, sex, BMI, and HIV status).

## Results

**Fig. 1:**
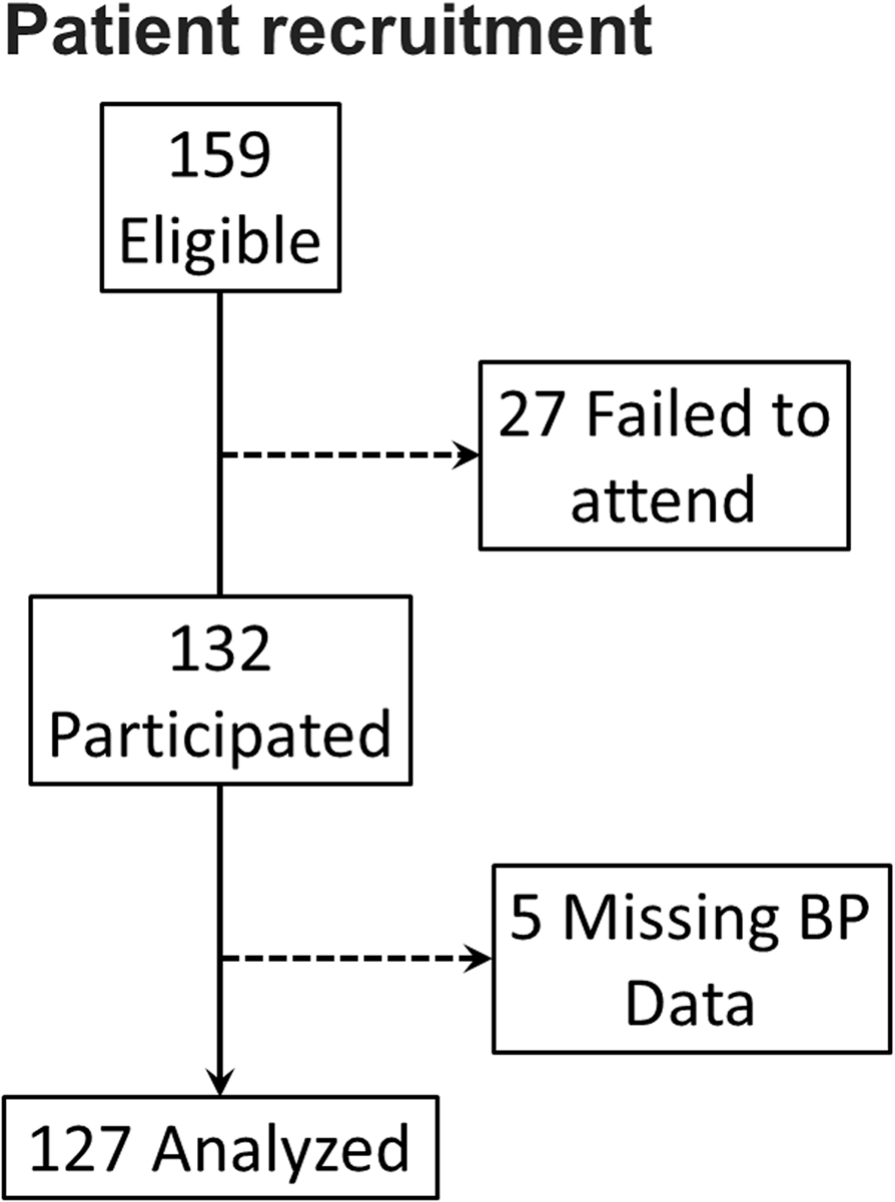
Patient recruitment flow diagram. BP, blood pressure

A total of 159 individuals were assessed for eligibility between December 2020 and March 2021 from which 27 were excluded after they missed their appointments. A total of 132 individuals completed the preintervention and postintervention but five were excluded due to missing BP values, leaving a total of 127 participants in the final analysis (Fig. 1).

The median age (interquartile range, IQR) of participants was 30 years (22–46 years) (Table 1). Among the total participants, 52% were female. The majority were unemployed (74%) and HIV-negative (80%). Median BMI was 23 (IQR, 21–27) and fasting blood sugar was 4.7 mmol/L (IQR, 4.2–5.1 mmol/L). Only 5% had an ABI lower than 0.9 suggesting peripheral artery disease. The largest differences between SBP and MAP produced by oral salt (minus baseline; median, IQR) in all subjects were 14 mmHg (7–21 mmHg) and 12 mmHg (7–16 mmHg), respectively (Table 1). A larger than expected proportion of participants (62%) were IPROS responders, whereas 32% of participants had increases in SBP that exceeded the usually considered clinically significant magnitude of 20 mmHg.

**Table 1:** Demographic and clinical characteristics of study participants

Characteristics	Value
Age (yr) (*n =* 127)	30 (22–46)
Body mass index (kg/m^2^) (*n =* 127)	23.1 (20.8–27.4)
Fasting blood sugar (mmol/L) (*n =* 127)	4.7 (4.2–5.1)
Sex (*n =* 127)	
Male	61 (48.0)
Female	66 (52.0)
Employment (*n =* 125)	
Employed	24 (19.2)
Unemployed	93 (74.4)
Retired	8 (6.4)
Marital status (*n =* 125)	
Married	46 (36.8)
Single	66 (52.8)
Divorced/separated	7 (5.6)
Widowed	6 (4.8)
HIV status (*n =* 125)	
Negative	100 (80.0)
Positive	25 (20.0)
Ankle brachial index (*n =* 125)	
Normal	119 (95.2)
Peripheral artery disease	6 (4.8)
∆SBP (mmHg), highest–baseline (*n =* 127)	14 (7–21)
∆MAP (mmHg), highest–baseline (*n =* 127)	12 (7–16)
IPROS (*n =* 127)	
Responder (MAP≥10 mmHg)	79 (62.2)
Nonresponder (MAP< 10 mmHg)	48 (37.8)
Other categories	
∆SBP ≥20 mmHg	40 (31.5)
∆SBP < 20 mmHg	87 (68.5)
RBC count ×10^12^/L (*n =* 127)	4.61 (3.91–5.09)
Absolute ESS (%) (*n =* 127)	140 (104–164)
ESS category (*n =* 117)	
Low (< 80%)	15 (12.8)
Average (80–120%)	27 (23.1)
High (> 120%)	75 (64.1)

Data are presented as median (interquartile range) or number (%)

*HIV* Human immunodeficiency virus, *∆SBP* Change in systolic blood pressure, *∆MAP* Change in mean arterial pressure, *MAP* Mean arterial pressure, *IPROS* Immediate pressor response to oral salt, *RBC* Red blood cell, *ESS* Erythrocyte sodium sensitivity

Median RBC count and median ESS were 4.61 (3.91–5.09) × 10^12^/L and 140% (104–164%), respectively. ESS was low, average, and high in 13, 23, and 64% of the 117 participants, respectively, in whom it was measured.

### Time course of blood pressures before and after the oral salt load

Table S1 shows the significant decreases of BP that occurred from arrival to the clinic until achieving a rested baseline 40 minutes later in all participants. We employed a long resting period because local conditions required long walking periods for many participants to reach the clinic.

**Fig. 2:**
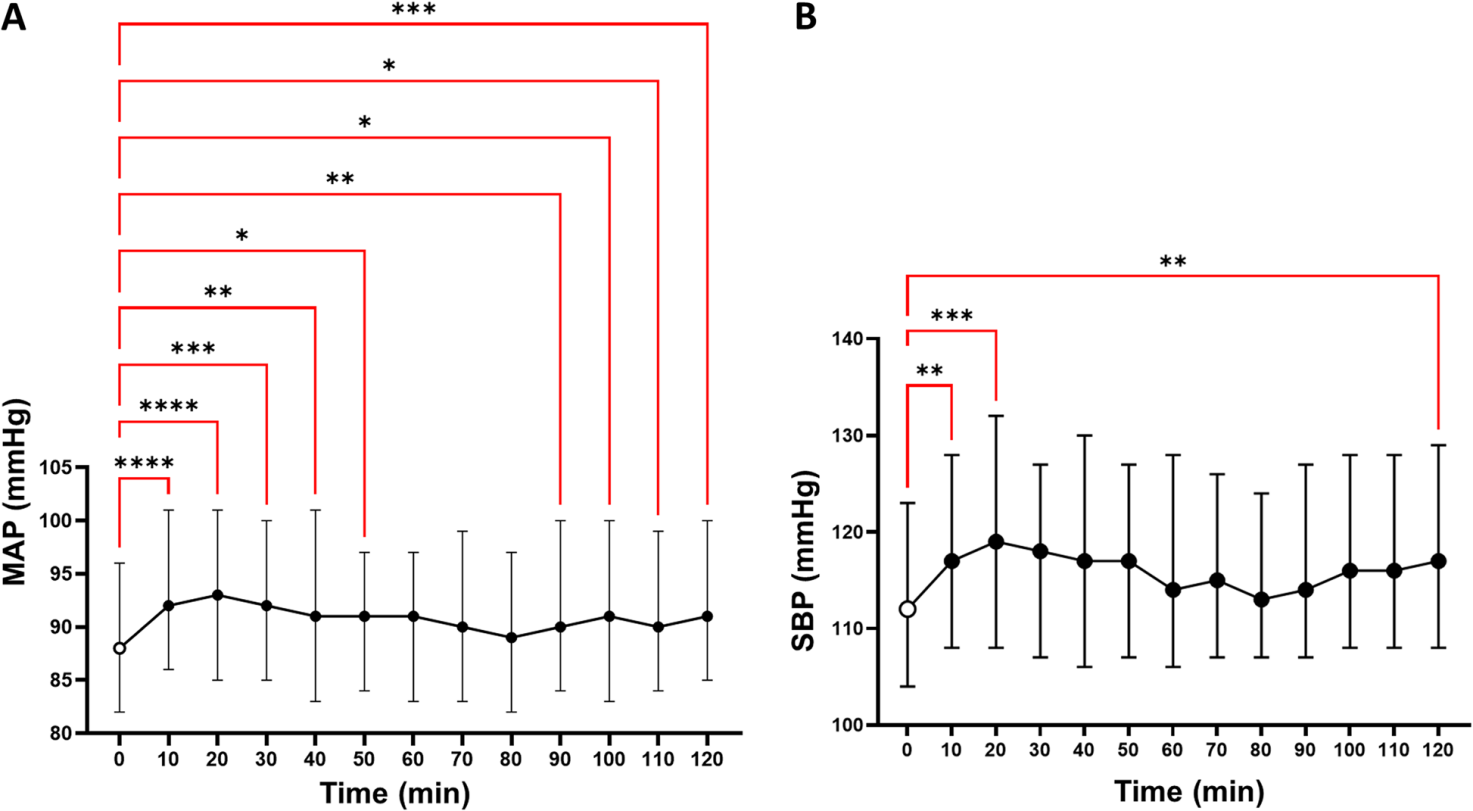
Blood pressures increase immediately after salt ingestion. Dots and vertical bars represent the median and interquartile range values for blood pressures measured every 10 minutes. The white dot, at time zero, represents the baseline blood pressure after a 40-minute period of resting. **A** After participants received 2 g of salt at time 0, mean arterial pressure (MAP) increased significantly at 10 minutes. Later, the course of MAP showed an apparent bimodal shape, as suggested by early and late significant increases over baseline (asterisks), with a mid-period (60 to 80 minutes) during which MAP almost returned to baseline. **B** Analogous patterns were observed for systolic blood pressure (SBP). The asterisks (Dunn’s multiple comparisons after a Freidman test) represent the following: *P < 0.05, **P < 0.01, ***P < 0.001, and ****P < 0.0001

Figure 2 shows that in all participants analyzed together, postintervention MAP (Fig. 2A) was significantly higher than baseline starting 10 minutes after the oral salt load, and its increase was sustained for the 120-minute duration of the study, with exception of the values at 60, 70, and 80 minutes, which although still higher than baseline, did not achieve statistical significance. The median difference between all postintervention MAPs and baseline was 3 (IQR, 2–4; *P <* 0.01). SBP (Fig. 2B) exhibited a similar pattern but the most significant changes were observed in the early and late recordings of the 120-minute period of study. The median difference between all postintervention SBPs and baseline was 4.5 mmHg (IQR, 3–5 mmHg; *P <* 0.001).

**Fig. 3:**
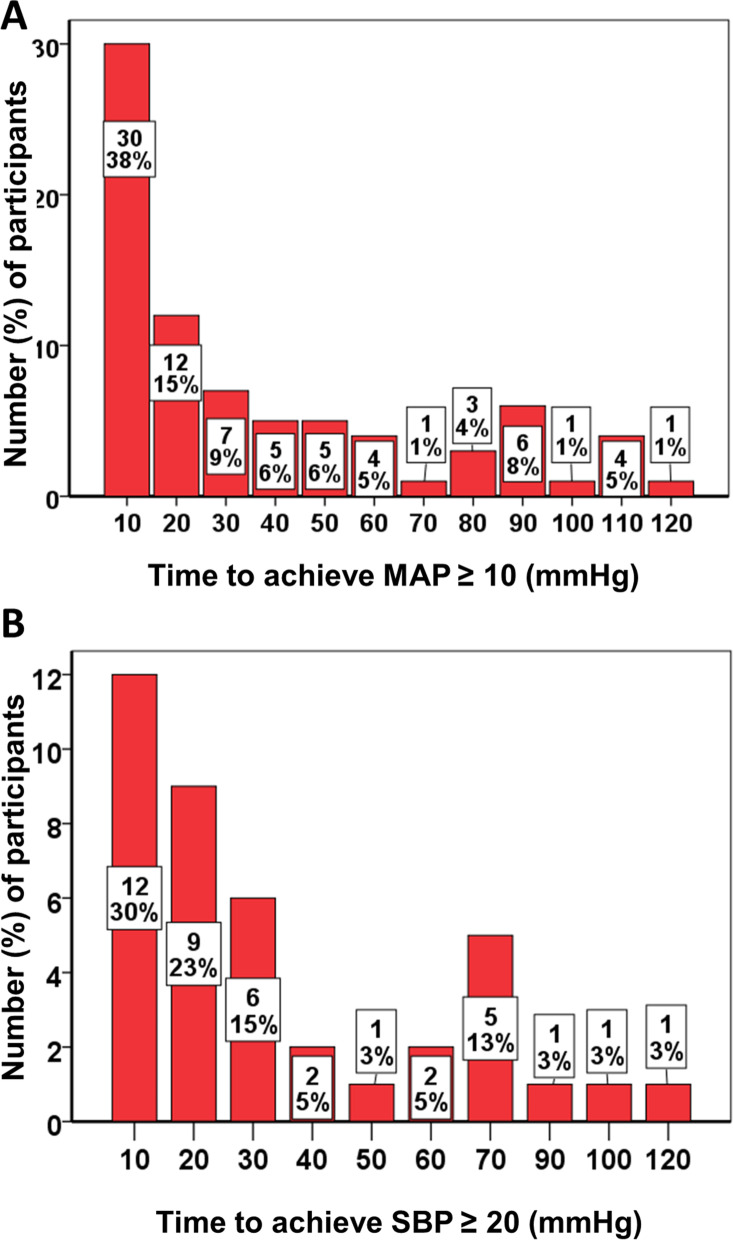
Proportion of participants exceeding blood pressure cutoffs at 10-minute time intervals, post-intervention. **A** Mean arterial pressure (MAP). **B** Systolic blood pressure (SBP)

Figure 3 shows the time intervals at which the participants with ∆MAP (Fig. 3A) and ∆SBP (Fig. 3B) of > 10 and > 20 mmHg (*n* = 79, and 78, respectively), actually exceeded those cutoffs. It can be seen that more than one third of IPROS responders (e.g., ∆MAP > 10 mmHg) can be identified within 10 minutes of the oral salt load. Within an hour, 79% of IPROS responders had exceeded the MAP cutoff, whereas 81% of the SBP responders had exceeded their respective cutoffs.

We used these raw proportions to rigorously assess the question of how many of the participants who will exceed BP cutoffs will do so at different intervals, with 95% confidence. Table S2 shows the results of this sample proportion summary hypothesis test. The lower 95% confidence limit for the proportion of participants who will exceed BP cutoffs surpassed 0.5 at 30 minutes for the two BPs (MAP and SBP). In other words, by 30 minutes after salt administration, half the responders will have responded, with 95% certainty. Analogously, by 60 and 90 minutes, about 80% (95% confidence interval [CI], 71–89; *P <* 0.001) and 92% (95% CI, 87–98; *P <* 0.001), respectively, achieved IPROS responder status. These results indicate that 90 minutes is sufficient to detect IPROS responder status within a confidence level of 87–98% using 2 g of sodium chloride taken with water.

### Prediction of IPROS by SBP readings in the clinic

MAP is rarely calculated in the routine clinic setting. Therefore, we investigated if and how SBP responses to the oral salt load relate to and predict IPROS responder status. Employing the Goodman and Kruskal’s gamma statistics, we found that SBP responses above and below the level of clinical significance were concordant with IPROS responses (*P <* 0.001), SBP and MAP responses related in positive fashion as expected (positive gamma), and that their association was strong (gamma approaching 1) (Table S3).

**Fig. 4:**
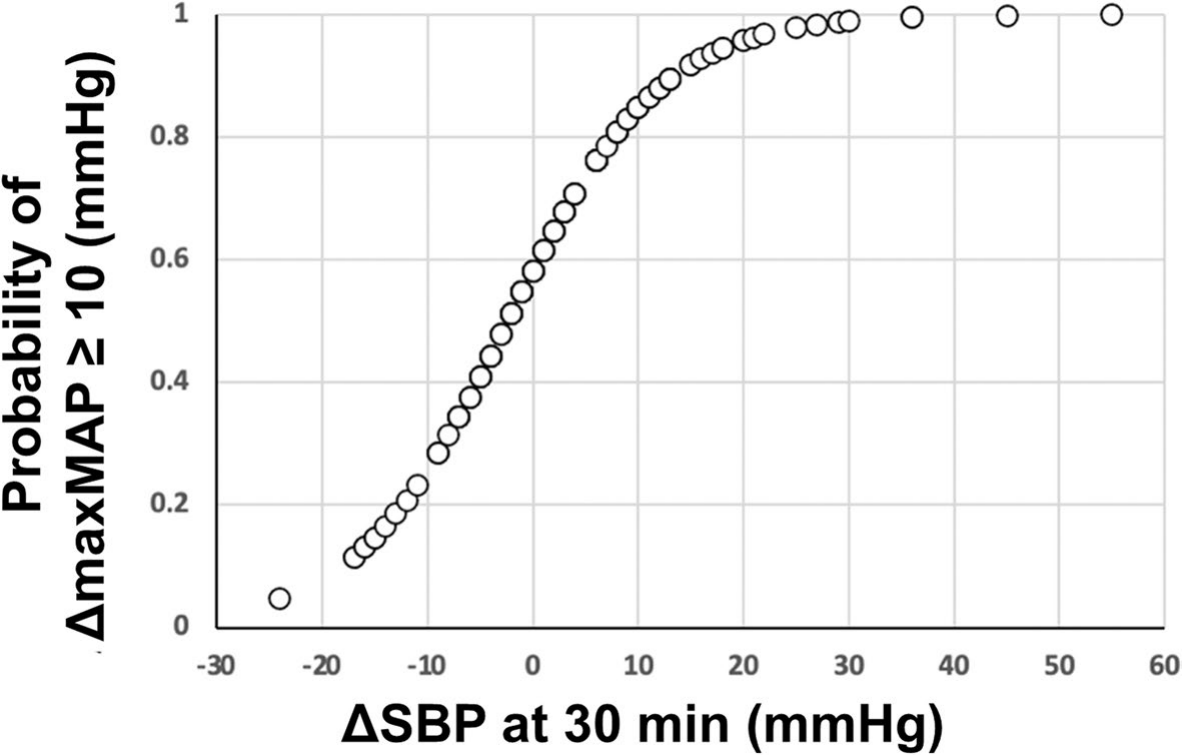
Immediate pressor response to oral salt responder predictive tool using ∆SBP at 30 minutes. The graph is obtained with a logistic regression. The X-axis shows ∆SBP at 30 minutes determining the probability of being a responder (Y-axis). ∆SBP, change in systolic blood pressure

In an age- and sex-controlled multivariable logistic regression, in which the dependent variable was a dummy for IPROS responder status and the regressors were the ∆SBPs (from baseline) at all 10-minute time intervals, the only significant predictor (adjusted odds ratio, 1.09; *P* = 0.026, not shown) was the ∆SBP 30 minutes after administration of salt. This result was reproduced using lesser numbers of ∆SBP time-interval combinations to optimize the ratio of number of observations per regressor (at least 15 to 1). We also confirmed that age and sex were not significant contributors to IPROS status in a reduced multivariable model in which the only included ∆SBP was the significant one at 30 minutes. Therefore, we proceeded to obtain the coefficients for the constant (0.3293) and for ∆SBP at 30 minutes (0.1391) in a univariable logistic regression between this variable and the IPROS dummy. With these coefficients we calculated the probability that a subject having a certain ∆SBP at 30 minutes is an IPROS responder with the formula *P* = 1 / 1 + e ^− (0.3293 + 0.1391 * ∆SBP30 min)^. Results are shown in Fig. 4 which can be used by the physician in the clinic as a prediction tool.

### Relationships between demographics and vascular function variables and IPROS

In univariate analyses, there were no detectable relationships between IPROS and demographic (age, sex, employment status, marital status, HIV status, and BMI) or clinical (fasting blood sugar) characteristics (Table 2).

**Table 2:** Factors associated with IPROS

Variable	IPROS responders *n =* 79 (62%)	IPROS nonresponders *n =* 48 (38%)	*P*-value
Age (yr)	29 (22–46)	31 (23–47)	0.707
Sex			
Male	38 (48.1)	23 (47.9)	0.594
Female	41 (51.9)	25 (52.1)	
Employment status (*n =* 125)			
Employed	14 (17.9)	10 (21.3)	0.899
Unemployed	59 (75.6)	34 (72.3)	
Retired	5 (6.4)	3 (6.4)	
Marital status (*n =* 125)			
Married	25 (32.1)	21 (44.7)	0.416
Single	43 (55.1)	23 (48.9)	
Divorced/separated	5 (6.4)	2 (4.3)	
Widowed	5 (6.4)	1 (2.1)	
HIV status (*n =* 125)			
Negative	62 (79.5)	38 (80.9)	0.854
Positive	16 (20.5)	9 (19.1)	
Body mass index (kg/m^2^)	23.4 (21.0–26.3)	23.0 (20.3–28.2)	0.933
Ankle brachial index category (*n =* 125)			
Normal	74 (94.9)	45 (95.7)	0.790
Peripheral artery disease	4 (5.1)	2 (4.3)	
Pulse pressure (mmHg)	2 (−6 to 11)	0 (−7 to 5)	0.040
Fasting blood sugar (mmol/L)	4.7 (4.2–5.1)	4.7 (4.2–5.0)	0.746
Erythrocyte sodium sensitive (*n =* 117)			
Low (< 80%)	11 (8.9)	4 (15.3)	0.004
Average (80–120%)	23 (8.9)	4 (31.9)	
High (> 120%)	38 (82.2)	37 (52.8)	

Data are presented as median (interquartile range) or number (%)

*IPROS* Immediate pressor response to oral salt, *HIV* Human immunodeficiency virus

**Fig. 5:**
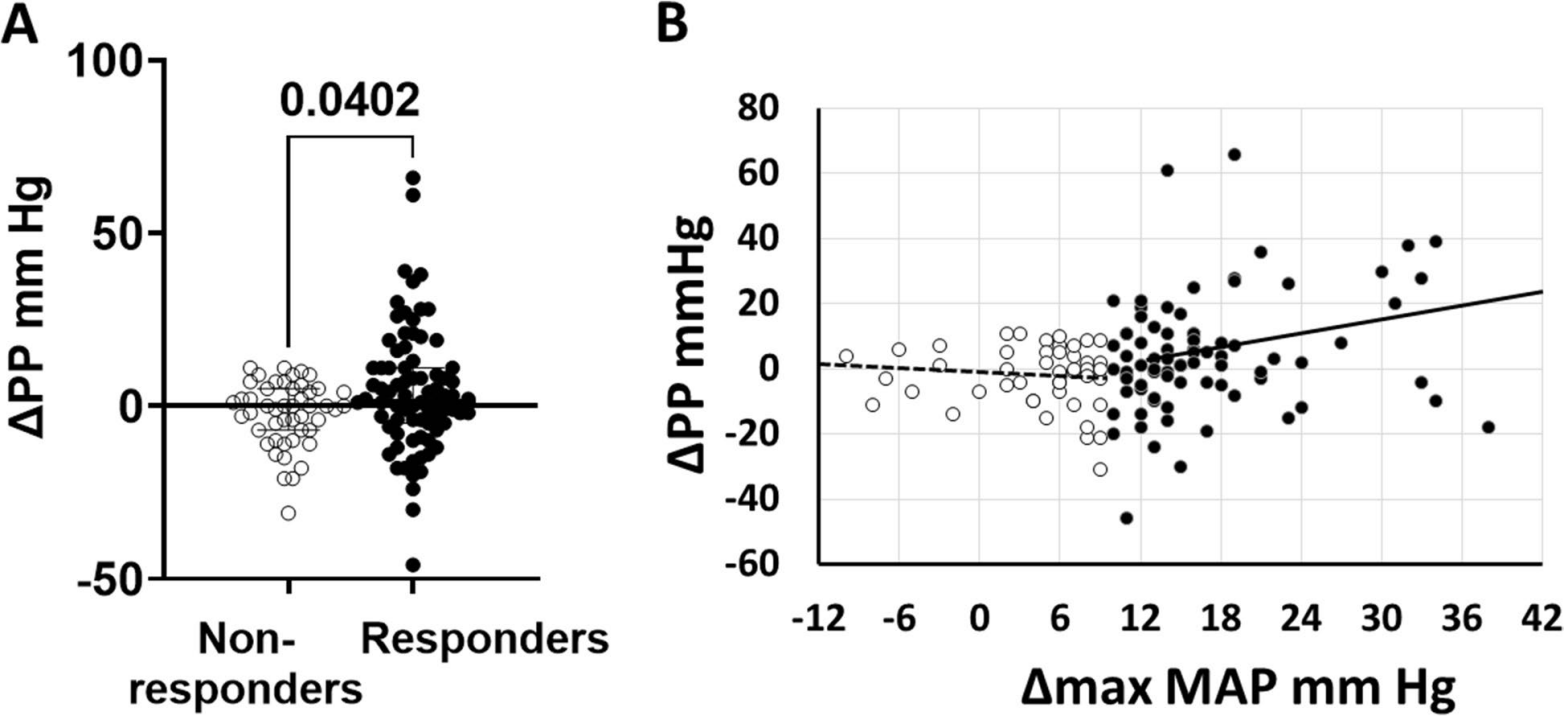
Simple linear regression between maximum change in pulse pressure (∆PP) and change in mean arterial pressure (∆MAP). **A** Responders had higher maximum change in pulse pressure compared to nonresponders. **B** A significant but weak relationship between the maximum individual mean arterial pressure response to salt (∆max MAP mmHg) and the concomitant change in pulse pressure (∆PP mmHg) in the entire population (*n =* 127, *r =* 0.26, *P <* 0.003, regression line not shown) was entirely attributable to the MAP responders (black dots and solid regression line, *n =* 79, *r =* 0.23, *P <* 0.04), not present in nonresponders (white dots and dashed line, *n =* 48, *r =* 0.10, not significant)

In terms of surrogate markers of vascular health, there was no difference in the proportion of normal or abnormal ankle brachial indices between IPROS responders and nonresponders (Table 2), an observation that is not surprising since 95.2% of these young healthy subjects had a normal test (i.e., narrow interindividual variability of results). In contrast, we found significantly higher pulse pressure responses to salt in IPROS responders (median, 2 mmHg; IQR, − 6 to 11 mmHg) than in nonresponders (median, 0; IQR, − 7 to 5; *P* = 0.040), at the time of maximal MAP responses in both groups. Inspection of these data in responders (Fig. 5A) suggest a skewed distribution, which was confirmed with a test for normality (Shapiro-Wilk) and explains the simple linear regression finding that change in pulse pressure (∆PP) and change in MAP (∆MAP) were correlated in responders (*r =* 0.23, *P <* 0.040) but not in nonresponders (Fig. 5B). Therefore, as opposed to findings on ABIs, those on pulse pressure suggest that acute changes in conduit artery elasticity may participate in the BP response to salt of the IPROS responders but not in nonresponder subjects, probably owing to acute changes in endothelial function [10].

In univariate analysis (Table 2), it appeared that a smaller percentage of subjects with low ESS was present among IPROS responders than in not responders, whereas the percentage of subjects with high ESS was also higher in the former, albeit not significant. Although these findings could also be taken to represent higher acute disruption of the endothelium by the salt load in IPROS responders, this apparent relationship was driven by confounders and was no longer present when we assessed the relationship between ESS and IPROS in multivariable logistic regression adjusted for age, sex, BMI, and HIV status (Table 3).

**Table 3:** Association between erythrocyte sodium sensitivity and IPROS in logistic regression

Erythrocyte sodium sensitivity status (%)	OR (95% CI)	P-value	Adjusted OR (95% CI)^a)^	*P*-value
Low (< 80)	1.00		1.00	
Average (80–120)	2.09 (0.43–9.96)	0.354	2.00 (0.42–9.79)	0.379
High (> 120)	0.37 (0.10–1.27)	0.117	0.33 (0.09–1.18)	0.089

*IPROS* Immediate pressor response to oral salt, *OR* Odds ratio, *CI* Confidence interval

^a)^ Adjusted for age, sex, body mass index, and human immunodeficiency virus status

## Discussion

Our research was aimed to study IPROS and its determinants in a normotensive Zambian population. Our study shows that almost two thirds of normotensive Zambian participants experienced significant immediate pressor responses to a modest salt intake, lasting for at least 2 hours. We do not know whether this is a specific finding among Africans, but this percentage is more than double the prevalence of traditional “salt sensitivity of BP” in other normotensive populations. In view of the evidence for a role of salt in the production of postprandial vascular dysfunction, which seems supported by pulse pressure data in our IPROS responders, the enhanced blood pressor response we observed may add to the BP-independent actions of sodium with potential detrimental clinical and prognostic significance.

Few investigators have measured the immediate effects of dietary salt on BP. Wahab et al. [21] reported significant increases in MAP and SBP 60 minutes after participants consumed a salty soup (4 g of sodium chloride). However, these increases were of small magnitude, assessed at one timepoint only, and without comparison of responder versus nonresponder participants. Nonetheless, the participants of this study exhibited worsening of endothelial (flow mediated dilatation) and vascular function (resistive and pulsatility indices of the carotid and renal arteries) 60 minutes after the salt load. The authors speculated that increased plasma sodium rather than BP may have accounted for their findings based on correlation analysis [22]. In another study by Dickinson et al. [10], endothelial function (assessed by brachial artery flow mediated relaxation), was also impaired 30 and 60 minutes after a high-salt meal (3.8 g) compared to a milder impairment produced by a low salt meal in the same healthy participants; this occurred without any significant change in BP. This demonstrates that an increase in dietary salt enhances the transient vascular dysfunction of the postprandial state.

### Time course response of BP to oral salt

No study has reported a time course assessment of the immediate response to oral salt that we are aware of. Assuming that the IPROS bears some relationship with SSBP defined by longer protocols [1, 3], and thus, shares its prognostic implications as supported by vascular dysfunction studies, our time course data have diagnostic implications because oral administration of salt with a 90-minute follow-up period is feasible in the routine clinic setting. Furthermore, the duration of the response, spanning the 120 minutes of our study, indicates that a responder who eats three salty meals per day increases the 24-hour BP load by having BPs higher than baseline for at least 6 hours or approximately 40% of the awake period, which is known to be of prognostic importance [23].

### SBP as a predictor of the IPROS

MAP, the integrated arterial pressure throughout the systolic and diastolic phases of the cardiac cycle, is determined by cardiac output and systemic vascular resistance and reflects tissue perfusion better than SBP and DBP [24, 25]. It has therefore been used in most studies of salt sensitivity of BP [1]. However, clinicians in practice rely mostly on SBP for diagnosis and therapeutic decisions. Our data indicate that the change in SBP 30 minutes after the salt load can be used to identify IPROS responders in the routine clinic setting.

### Relationships between vascular function variables and IPROS

We chose pulse pressure, ABI, and ESS data as surrogate markers for conduit artery elasticity and vascular endothelial dysfunction because they are noninvasive, easy to obtain in the clinic and inexpensive. Our hypothesis was that these markers would be impaired in IPROS responders, consistent with the rapid induction of postprandial endothelial dysfunction by a high-salt meal described by others [10, 21]. Increased pulse pressure detects stiffening of conduit arteries, decreased ABI detect abnormalities in conduit or smaller arteries of the lower extremities, whereas increased ESS reflects more generalized disruption of the endothelial glycocalyx. The only marker that related to IPROS was the pulse pressure response to salt, and this relationship was only present in IPROS responders, suggesting that acute changes in endothelial function may occur in their conduit arteries and be responsible, at least in part, for the overall acute pressor response to salt. The fact that the observation for ABIs was not significant is most likely accounted for the normal result of this test in more than 95% of our normotensive healthy participants, thus lacking enough variability to detect relationships. In contrast, the lack of relationship between ESS and IPROS is more difficult to explain, particularly in the presence of that between pulse pressure in IPROS. Perhaps ESS detects only late, generalized, and cumulative effects of salt on structural changes of the glycocalyx over time, which may not have yet developed in our young IPROS responders, but this is merely a speculation.

### Novelty

To our knowledge, this is the first study to investigate the immediate arterial response to oral salt in a defined population and to describe its time course and its prediction by concomitant SBP, which is the BP used by clinicians.

### Clinical implications

IPROS in blacks is not well reported but BP perturbations in general are a risk factor for future development of hypertension. Specifically, SSBP in normotensives (assessed by dietary or by the rapid intravenous Weinberger salt load protocol), predicts the development of incident hypertension. We hope to pursue long-term follow-up of our population and thus assess whether IPROS exerts an analogous predictive role.

It has been previously shown that salt acutely induces endothelial dysfunction above and beyond that produced by the postprandial state and that this effect of salt is independent of BP changes. We now show that at least in our population, there is a significant pressor response to oral salt in a large percentage of black participants, despite having defined such response with very stringent criteria. We also provide preliminary findings about the effect of salt on pulse pressure, which are suggestive of an acute effect of salt on conduit artery elasticity, perhaps mediated by acute effects on endothelial function, as reported by others [10]. We suspect that the combination of acute salt-induced endothelial dysfunction and BP elevation, with the latter increasing 24-hour BP load owing to its duration, is also a risk factor candidate for cardiovascular disease, mimicking that of traditionally defined salt sensitivity of BP [9], a contention that remains to be proven.

### Limitations

Some important limitations of our study include: (1) We did not include hypertensive individuals in whom the immediate pressor response to salt may be qualitatively or quantitatively different from that of healthy participants. The study was conducted on young and middle age normotensive participants with a relatively narrow-age range for three reasons. First, due to the location of our hospital and its referring center role, subjects walk long distances and wait in long queues to be seen. The elderly avoid these hardships by visiting health facilities closer to their homes. Second, our center is responsible for issuing health certificates to applicants for government employment and driving licenses, an usually youthful healthy population. Third, we are aware that pressor responses to salt are confounded by already established hypertension (e.g., prevalence of SSBP increases from 25 to 30% in normotensive to ≥50% in Western and African populations [14, 15]) and that the putative genetic susceptibility to the pressor effect of salt has mostly been studied in young populations without hypertension [26]. In recruiting normotensive subjects for these reasons, we certainly ended up with a relatively young population and this is important because in Africa, as in the Western world, the prevalence of hypertension increases with aging [27, 28]. Therefore, our findings are only generalizable to normotensive adults in a narrow-age range. (2) Our population is known to have high prevalence of traditionally defined salt sensitivity of BP, something that we did not attempt to measure; therefore, the relationship between IPROS and such salt sensitivity remains unknown. (3) We did not control for pre-study dietary intake of salt beyond the protocol mandated 8-hour fasting period, which may have influenced IPROS results. (4) We did not measure urine sodium excretion before or during the protocol, agreeing with the recommendation by the World Hypertension League that deems short term urine collections a dismal representation of sodium intake or excretion, which should not be pursued or reported [29]. This contention is made stronger by the observation that even in a fully controlled environment a minimum of 7-day measurements of sodium excretion is required to estimate a real-life unachievable stable sodium intake owing to the existence a circaseptan rhythm [30] making even a 24-hour urine collection not a “gold standard” for assessment of salt intake. Also, it is well known that salt resistant and salt sensitive humans do not differ in the handling of a salt load, but only in that the latter require BP elevation to achieve salt balance whereas the former do not [1]. Therefore, although we discuss the issue of urine sodium measurement in this section, we believe that this is not a limitation of our study. (5) Surrogates of vascular and endothelial dysfunction provided discordant results, an issue that remains to be studied with techniques that detect more subtle vascular damage or with studies with long-term follow-up of a significantly large population to assess actual outcomes. Despite its potential limitations, our study provides a characterization of the prevalence and time course of IPROS, its determinants in a young normotensive population, and the tool for its practical assessment in the clinic setting, all novel features for understanding immediate effects of salt on cardiovascular health.

## Conclusions

We confirm the presence of an immediate and significant pressor response to oral salt in a high proportion (62%) of otherwise normotensive participants and describe its time course and correlates. Although the IPROS did not correlate with sociodemographic and clinical factors, SBP 30 minutes after the oral moderate salt load was a good predictor of responder status. The average time course to achieve an IPROS was 30 minutes, and the increase was sustained for the 120-minute duration of the study. This time course is important in informing clinical studies assessing the immediate effects of salt on vascular function. In addition to its known effects on postprandial vascular dysfunction, perhaps supported by our data on pulse pressure, our study indicates that oral salt may increase the 24-hour BP load, which would make it a risk factor for developing incident hypertension and perhaps cardiovascular morbidity. Our findings are therefore relevant for clinical practice, indicating the need to include dietary sodium assessments in monitoring BP and in the diagnosis, prevention, and management of hypertension.

## Supplementary Information

**Additional file 1 Table S1.** Preintervention blood pressures over 40 minutes, from onset of experiment until baseline. **Table S2.** Proportion of participants with MAP and SBP exceeding cutoffs at each time interval. **Table S3.** Strength of association between IPROS and BP response categories

## Data Availability

All data generated or analyzed during this study are included in this published article. For other data, these may be requested through the corresponding author.

## Abbreviations

ABI: Ankle brachial index
BMI: Body mass index
BP: Blood pressure
CI: Confidence interval
CVD: Cardiovascular disease
DBP: Diastolic BP
ESS: Erythrocyte sodium sensitivity
IPROS: Immediate pressor response to oral salt
IQR: Interquartile range
MAP: Mean arterial pressure
RBC: Red blood cells
SBP: Systolic BP
SSBP: Salt sensitivity of BP
