# Supplementary material for: Immediate pressor response to oral salt and its assessment in the clinic: a time series clinical trial
Source: Clin Hypertens. 2022 Sep 15;28:25. doi: 10.1186/s40885-022-00209-2 (PMC9476589; doi:10.1186/s40885-022-00209-2)
Supplement: Supplementary file 1 — Additional file 1 Table S1. Preintervention blood pressures over 40 minutes, from onset of experiment until baseline. Table S2. Proportion of participants with MAP and SBP exceeding cutoffs at each time interval. Table S3. Strength of association between IPROS and BP response categories [file 40885_2022_209_MOESM1_ESM.docx]

**Table S1.** Preintervention blood pressures over 40 minutes, from onset of experiment until baseline

| Variable | –40 min | –30 min | –20 min | –10 min | Baseline^a)^ | P-value |
| --- | --- | --- | --- | --- | --- | --- |
| MAP (mmHg) |  |  |  |  |  |  |
| Median (IQR) | 93 (88–105) | 92 (85–100) | 92 (85–100) | 91 (84–98) | 88 (82–96) | <0.001 |
| 95% CI | 93.4–97.9 | 91.5–95.8 | 90.7–94.9 | 90.5–94.6 | 87.6–91.6 |  |
| Adjusted P-value | <0.001 | <0.001 | <0.001 | <0.001 | N/A |  |
| SBP (mmHg) |  |  |  |  |  |  |
| Median (IQR) | 119 (110–133) | 118 (110–127) | 117 (108–129) | 116 (108–126) | 112 (104–123) | <0.001 |
| 95% CI | 119–125 | 117–123 | 116–122 | 115–121 | 113–118 |  |
| Adjusted P-value | <0.001 | <0.001 | <0.001 | <0.001 | N/A |  |

Time headings indicate minutes before baseline (salt administration). P-values in rightmost column are for the Friedman test. Adjusted P-values in rows are for post-hoc Dunn’s multiple comparison.

MAP, mean arterial pressure; IQR, interquartile range; CI, confidence interval; N/A, not applicable; SBP, systolic blood pressure.

^a)^Baseline blood pressures are the average of three recordings.

**Table S2.** Proportion of participants^a)^ with MAP and SBP exceeding cutoffs at each time interval

| Time taken to respond (min) | MAP ≥10 mmHg, n = 79  n (proportion) | 95% CI | P-value | SBP ≥20 mmHg, n = 40  n (proportion) | 95% CI | P-value |
| --- | --- | --- | --- | --- | --- | --- |
| 10 | 30 (0.38) | 0.27–0.49 | 0.980 | 12 (0.30) | 0.16–0.44 | 0.990 |
| 20 | 42 (0.53) | 0.42–0.64 | 0.280 | 21 (0.53) | 0.37–0.68 | 0.370 |
| 30 | 49 (0.62) | 0.51–0.72 | 0.016 | 27 (0.68) | 0.52–0.82 | 0.013 |
| 40 | 54 (0.68) | 0.58–0.78 | 0.006 | 29 (0.73) | 0.59–0.86 | 0.002 |
| 50 | 59 (0.75) | 0.65–0.85 | <0.001 | 30 (0.75) | 0.62–0.88 | 0.008 |
| 60 | 63 (0.80) | 0.71–0.89 | <0.001 | 32 (0.80) | 0.68–0.92 | <0.001 |
| 70 | 64 (0.81) | 0.72–0.90 | <0.001 | 37 (0.93) | 0.84–1.01 | <0.001 |
| 80 | 67 (0.85) | 0.77–0.93 | <0.001 | 37 (0.93) | 0.84–1.01 | <0.001 |
| 90 | 73 (0.92) | 0.87–0.98 | <0.001 | 38 (0.95) | 0.88–1.02 | <0.001 |
| 100 | 74 (0.94) | 0.88–0.99 | <0.001 | 39 (0.98) | 0.93–1.02 | <0.001 |
| 110 | 78 (0.99) | 0.96–1.01 | <0.001 | 39 (0.98) | 0.93–1.02 | <0.001 |
| 120 | 79 (1.00) | N/A | <0.001 | 40 (1.00) | NA | <0.001 |

MAP, mean arterial pressure; SBP, systolic blood pressure; CI, confidence interval; NA, not applicable**.**

^a)^Proportions estimated with sample proportion summary hypothesis testing.

**Table S3.** Strength of association between IPROS and BP response categories

| Variable | IPROS | | Gamma | P-value |
| --- | --- | --- | --- | --- |
|  | Responder  (n = 79) (62.2%) | Nonresponder  (n = 48) (37.8%) |  |  |
| Systolic BP change |  |  | 0.957 | <0.001 |
| <20 mmHg | 40 (50.6) | 47 (97.9) |  |  |
| ≥20 mmHg | 39 (49.4) | 1 (2.1) |  |  |

Data are presented as number (%).

IPROS, immediate pressor response to oral salt; BP, blood pressure.
